# Diagnosis and Management of an Inferior ST-elevation Myocardial Infarction: A Simulation Scenario

**DOI:** 10.7759/cureus.3995

**Published:** 2019-02-01

**Authors:** Desmond Whalen, Cody Dunne, Adam Dubrowski, Liban Mohamed, Michael H Parsons

**Affiliations:** 1 Emergency Medicine, Memorial University of Newfoundland, St. John's, CAN; 2 Medical Education and Simulation, Memorial University of Newfoundland, St. John's, CAN; 3 Internal Medicine, Memorial University of Newfoundland, St. John's, CAN

**Keywords:** simulation, emergency medicine, canmeds

## Abstract

Immediate diagnosis and management of ST-elevation myocardial infarction (STEMI), a condition resulting from the complete occlusion of a coronary artery, is critical to achieving optimal patient outcomes. This report outlines an acute inferior STEMI simulation which can be used for teaching different levels of learner including novice, intermediate and advanced. It focuses on the presentation, diagnosis, and management of inferior myocardial infarctions. Additionally, it incorporates the advanced cardiovascular life support (ACLS) protocol for more advanced learners and uses the CanMEDS collaborator/communicator role as an adjunct objective for all learners.

## Introduction

Chest pain is a common presentation of a life-threatening condition known as acute ST-elevation myocardial infarction (STEMI). Accurate recognition and assessment of this condition is essential as the 30-day mortality of myocardial infarctions and unstable anginas (collectively acute coronary syndrome) may reach 10% [1]. Myocardial infarctions occur when there is occlusion of coronary vessels, resulting in ischemia, and eventually myocardial necrosis [2]. Death of myocardial tissue can result in devastating complications such as heart failure, cardiogenic shock, cardiac arrest, and patient mortality [3].

In the context of emergency medicine, acute chest pain of cardiac origin is described by a broader term known as acute coronary syndrome (ACS) [4]. STEMI typically presents with a history of substernal or left-sided chest discomfort that may radiate into the arm, neck, jaw, back, abdomen, or shoulder [4]. However, a broad spectrum of atypical presentations may also occur. ST-segment elevation of greater than 1 mm (greater than 2 mm in leads V1/V2 in males) in two anatomically contiguous leads on an electrocardiogram (ECG) in the patient with fitting history and physical exam findings are adequate for diagnosis in an acute setting [3]. Later elevation of cardiac biomarkers such as Troponin T or Troponin I correlates with the diagnosis of a STEMI [5].

Inferior STEMI is usually caused by occlusion of the right coronary artery, or less commonly the left circumflex artery, causing infarction of the inferior wall of the heart [6, 7]. Upon ECG analysis, inferior STEMI displays ST-elevation in leads II, III, and aVF. There are subtle differences in the ECG pattern depending on the artery occluded. Reciprocal changes (ST-segment depression) may be seen in lead aVL [6]. ST-elevations in additional leads V4R or V7-V9, will indicate the presence of right ventricular and posterior involvement, respectively [6, 7]. Prompt recognition of inferior STEMI is essential as it will influence the type and efficacy of therapy provided. For instance, the beneficial effects of reperfusion therapy are highest when started soon after clinical presentation [3].

This technical report is designed to educate the medical students, residents and attending physicians in all levels of training at Memorial University of Newfoundland.

## Technical report

**Figure 1:**
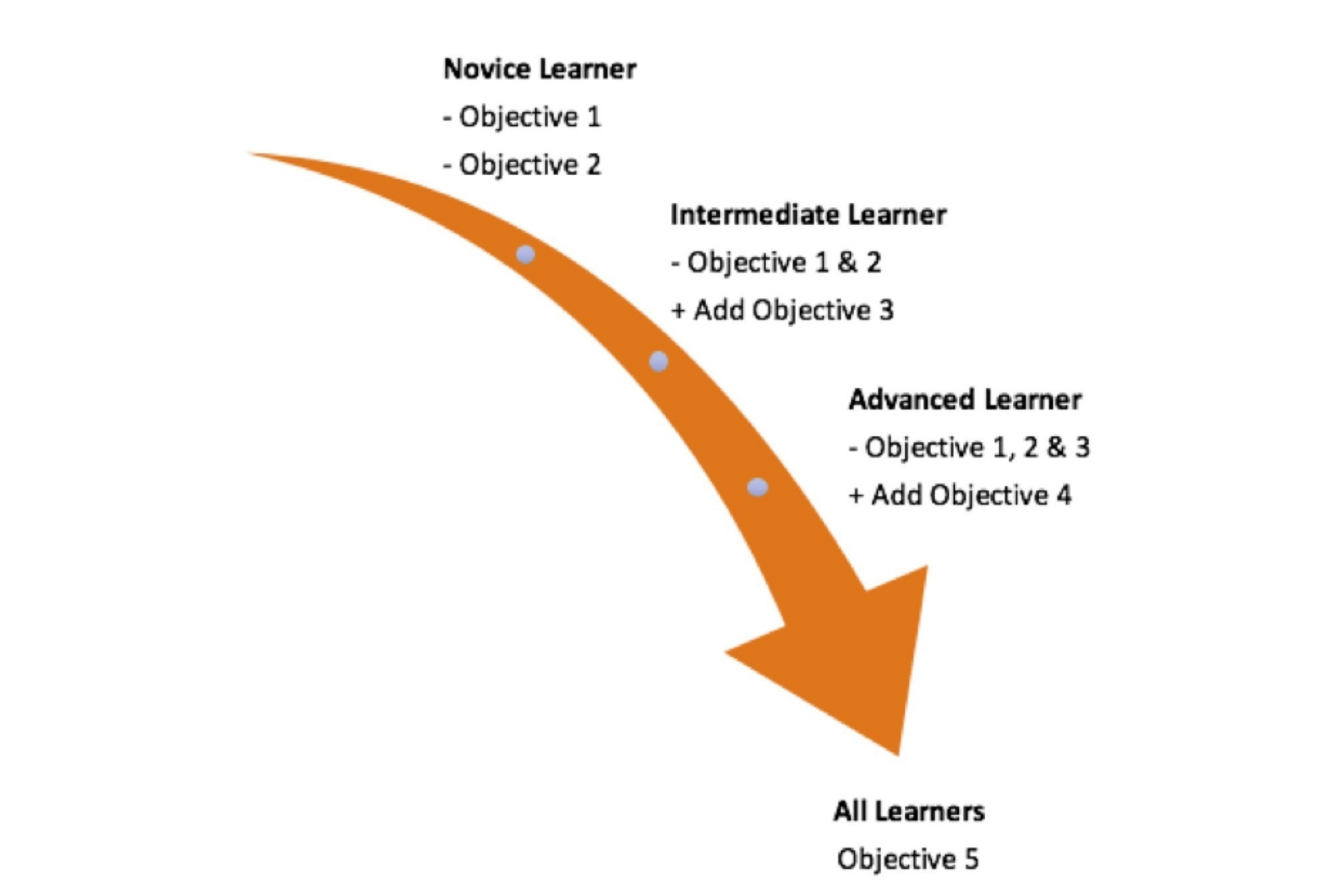
Application of case objectives as relevant to different learners.

The objectives of this study are outlined below, and Figure 1 illustrates how these objectives could build upon each other in a competency-based training program:

For the novice learners:

Objective 1: Initial management of ACS

Objective 2: Make the diagnosis of inferior STEMI and carry out initial management

For the intermediate learners, objectives include the above in addition to:

Objective 3: Managing complications

For advanced learners, objectives include the above in addition to:

Objective 4: Managing pulseless rhythms using ACLS

For all learners, the CanMEDS communicator and collaborator roles can be incorporated with the following objective:

Objective 5: Structuring a patient handover

This scenario takes place in a simulated emergency room of a community hospital or a tertiary care centre depending on the instructor’s preference and the desire to have interventionalists available on site. The room setting is a resuscitation bay with a full complement of resuscitation cart, defibrillator, and difficult airway equipment available. Drugs necessary for advanced cardiac life support and rapid sequence intubation are also provided. The patient is the Laerdal 3G human patient simulator.

Prior to this session, a stepwise, detailed scenario template was developed. The template included all relevant clinical data, radiological, ECG, and laboratory results that were necessary for the scenario’s execution. The template was developed and submitted to the simulation lab technical staff who programmed the mannequin and supplied the required material for the scenario’s execution.

To ensure a smooth experience for trainees, an instructor completed a run-through of the scenario while acting as a trainee prior to formal learning sessions. During the learning session, two instructors were present, one who maintained overall control of the scenario, and the second who took notes/completed grading schemes for the subsequent debriefing.

Pre-briefing

A pre-briefing is held with the trainees before the case. Of the learners present, those who will participate directly in the scenario are identified. The scenario leader is assigned or agreed upon among the learners. General limitations to be expected in the scenario are identified. Any technical issues with the mannequin and limitations with resource availability are reviewed. The fiction contract – the agreement between participants and instructors to proceed as if the simulation is real while simultaneously acknowledging it is not – is reviewed with all the trainees and instructors. In general terms, the source and rationale for including the case in the curriculum is discussed. Finally, the trainees are advised of the nature of assessment for the case (formative, summative, strictly practice, etc.).

Case

In this simulation case, a 51-year-old patient presents with two hours of epigastric pain that is not relieved by antacid treatment. Upon their request, trainees are provided with details of the patient’s allergies, medications, past medical history, and family history.

At the beginning of the scenario, the patient is connected to cardiac monitors with a full set of vital signs provided for junior learners. For more advanced learners, the scenario begins with the patient just placed in the bed and participants must request for monitors to be connected. Table 1 outlines the stepwise, detailed scenario used to program the mannequin by technical staff.

**Table 1:** A stepwise, detailed scenario used to program mannequin by technical staff.

Pre-Scenario Information		
You are an emergency room physician in a community hospital (or tertiary care centre). There are some specialty backup services available (on-site or by telephone). A 51-year-old patient presents with two hours of epigastric pain that is not relieved by antacids.		
History		
History of Presenting Illness	Pain began while out walking. The patient has had similar “heartburn” type symptoms over the past few weeks when working in the yard or walking up stairs. Today the pain is not subsiding despite taking antacids. The pain is retro-sternal, non-radiating, and is associated with shortness of breath, nausea and diaphoresis.	
Allergies	None	
Medications	Ranitidine and Insulin	
Past Medical History	Gastroesophageal reflux disease (GERD) and insulin dependent diabetes mellitus	
Social History	1 pack per day smoker x 30 years and occasional alcohol use	
Family History	Brother had a myocardial infarction (MI) at age 52 and has “stents”	
Review of Symptoms	Short of breath, nauseated, feels uncomfortable and anxious.	
Physical Exam		
General	Diaphoretic. The patient appears uncomfortable.	
Initial Vitals	Temperature (T) 37.0 C axillary / Heart Rate (HR) 55 / Blood Pressure (BP) 90/50 / Respiratory Rate (RR) 20 / Oxygen Saturation (SpO_2_) 96% on room air	
Head, Eyes, Ears, Nose, Throat (HEENT)	Nil	
Central Nervous System (CNS)	Oriented to person, place, and time	
Chest	Air entry equal bilaterally. Clear chest.	
Cardiovascular System (CVS)	Bradycardia. No murmurs. Mild jugular vein distension (JVD)	
Abdomen	Soft and non-tender, bowel sounds present.	
Objective 1: Initial Management of Acute Coronary Syndrome (ACS)		
Place the patient on telemetry (if not already completed by staff)		
Obtain Intravenous (IV) access		
Administration of O_2_ via nasal prongs (optional due to current level of oxygen saturation)		
Order 12-lead electrocardiogram (ECG) within two minutes after case starts (Figure 2)		
Order labs: complete blood count, creatine kinase, lactate dehydrogenase, liver function test, Troponin & coagulation studies		
Administer IV Normal Saline Bolus (500-1000cc)		
Administer Acetylsalicylic acid (ASA) 160 milligram chewable per oral (PO)		
Order Portable Chest X-ray (CXR) (Figure 3)		
Objective 2: Making the Diagnosis and Initial Management of Inferior STEMI		
Stage	Vitals/ Patient Status/ Findings	Expected Action
If appropriate initial actions taken	T 37.0°C axillary / HR 43 / BP110/70 / RR16 / SpO2 97%; transient BP response	Ask for ECG results
If fluids not administered	T 37.0°C axillary / HR40 / BP70/40 / RR24 / SpO2 93%; drowsy and more nauseated	Administer IV Normal Saline Bolus (500-1000cc) - Ask for repeat ECG
12-Lead ECG	Recognizes inferior STEMI	Order 15-lead ECG- rule out posterior STEMI and assess for further evidence of right ventricular involvement (Figure 4)
The results of ordered tests	ECG – Sinus Bradycardia with inferior STEMI findings (See Figure 2) Portable CXR- unremarkable	Identify bradycardia, history, and ECG findings and consolidate all factors together to make diagnosis of an inferior STEMI.
Proper identification of Inferior STEMI with appropriate initial actions taken (with specialist direction for novice +/- intermediate learners)	T 37.0°C axillary / HR55 / BP90/70 / RR16 / SpO2 95%; transient BP response	1) Administer vasopressor agent/atropine; 2) Administer platelet aggregation inhibitor* (for example): Plavix 300 mg PO; Plavix 600 mg PO if cath lab on site; 3) Administer anticoagulants* (for example): Heparin ACS protocol Enoxaparin 1 mg/kg subcutaneous (SC). Note that specific medications may depend on local availability and consultant preference
If expected actions not taken	T 37.0°C axillary / HR30 / BP70/40 / RR16 / SpO2 92%; patient less responsive	Take expected actions above + fluid bolus
If nitroglycerin, beta-blockers or morphine given	T 37.0°C axillary / HR30 / BP 60/40 / RR16 / SpO2 93%; patient less responsive	Take expected actions above + fluid bolus
If appropriate actions taken and catheterization (cath) lab IS available on Consult	T 37.0°C axillary / HR80 / BP110/70 / RR18 / SpO2 95%	Consult cardiology EARLY for assistance on treatment plan and proceed to End Scenario of catheterization lab
If appropriate actions taken and cath lab IS NOT available on Consult	T 37.0°C axillary / HR80 / BP110/70 / RR18 / SpO2 95%	1) Consult cardiology and express concern for cardiogenic failure; 2) Administer thrombolytic treatment: Tenecteplase (TNK) (weight-based dosing) & Enoxaparin 30 mg IV
End Scenario for Novice Learners or proceed to Objective 5		
Objective 3: Managing Complications (performance of procedures including intubation, transvenous pacing reserved for advanced learners)		
Stage	Vitals/ Patient Status/ Findings	Expected Actions
Patient deteriorates following lytic treatment (drowsier; bradycardia)	T 37.0°C axillary / HR30 / BP70/50 / RR12 / SpO2 90%	1) Consider intubation (call respiratory therapist or anesthesia if on-site); 2) Trial of transcutaneous pacing
Trial of transcutaneous pacing attempted	T 37.0°C axillary / HR30 / BP70/50 / RR12 / SpO2 90-92%; poor capture and the patient uncomfortable	1) Recognize no capture of transcutaneous pacing; 2) Prepare for transvenous pacing
Preparation for transvenous pacing	T 37.0°C axillary / HR70 / BP110/80 / RR12 / SpO2 90-92%	Proceed to end scenario (for intermediate)
End Scenario for Intermediate Learners or proceed to Objective 5		
Objective 4: Managing Pulseless Rhythms using Advanced Cardiac Life Support (ACLS)		
Stage	Vitals/ Patient Status/ Findings	Expected Actions
Pulseless ventricular tachycardia OR ventricular fibrillation	T 37.0°C axillary / HR not palpable / BP absent / RR agonal / SpO2 not detected	Cardiopulmonary Resuscitation (CPR) started and appropriate treatment of rhythm according to ACLS protocol
Proper ACLS Protocol Followed - Return of Spontaneous Circulation (ROSC)	T 37.0°C axillary / HR40 / BP 90/50 / RR10 / SpO2 90-95% assisted on 100% Bag-Valve-Mask	Post ROSC Care including: 1) Consider cooling therapy; 2) Definitive Airway; 3) End-tidal carbon dioxide monitoring (EtCO2); 4) Transfer to Cardiovascular Intensive Care Unit (CVICU)
Incorrect ACLS Protocol Followed	T 37.0°C axillary / HR not palpable / BP absent / RR agonal / SpO2 not detected	1) Start proper ACLS Algorithm; 2) Secure airway. It is important for instructors to decide beforehand whether or not discussion of death will be a specific outcome of the case and a point of discussion during post-scenario debriefing
End Scenario for Advanced Learners		
Objective 5: Structuring a Patient Handover		
Proper Consultation with Interventionist or Cardiologist	T 37.0°C axillary / HR70 / BP110/80 / RR20 / SpO2 97% NRB	Use structured way of presenting case, for example SBAR (Situation Background Assessment Recommendation) (Table 3)
End Scenario for Novice Learners		
Scenario Conclusion (Endpoints)		
Stabilization and transfer if: 1) Appropriate initial actions are taken; 2) Inferior STEMI is properly identified; 3) Appropriate agents are administered; 4) Definitive management is available; 5) Proper ACLS Protocol is followed (for Advanced Learners); 6) Proper hand-over to definitive management		

**Figure 2:**
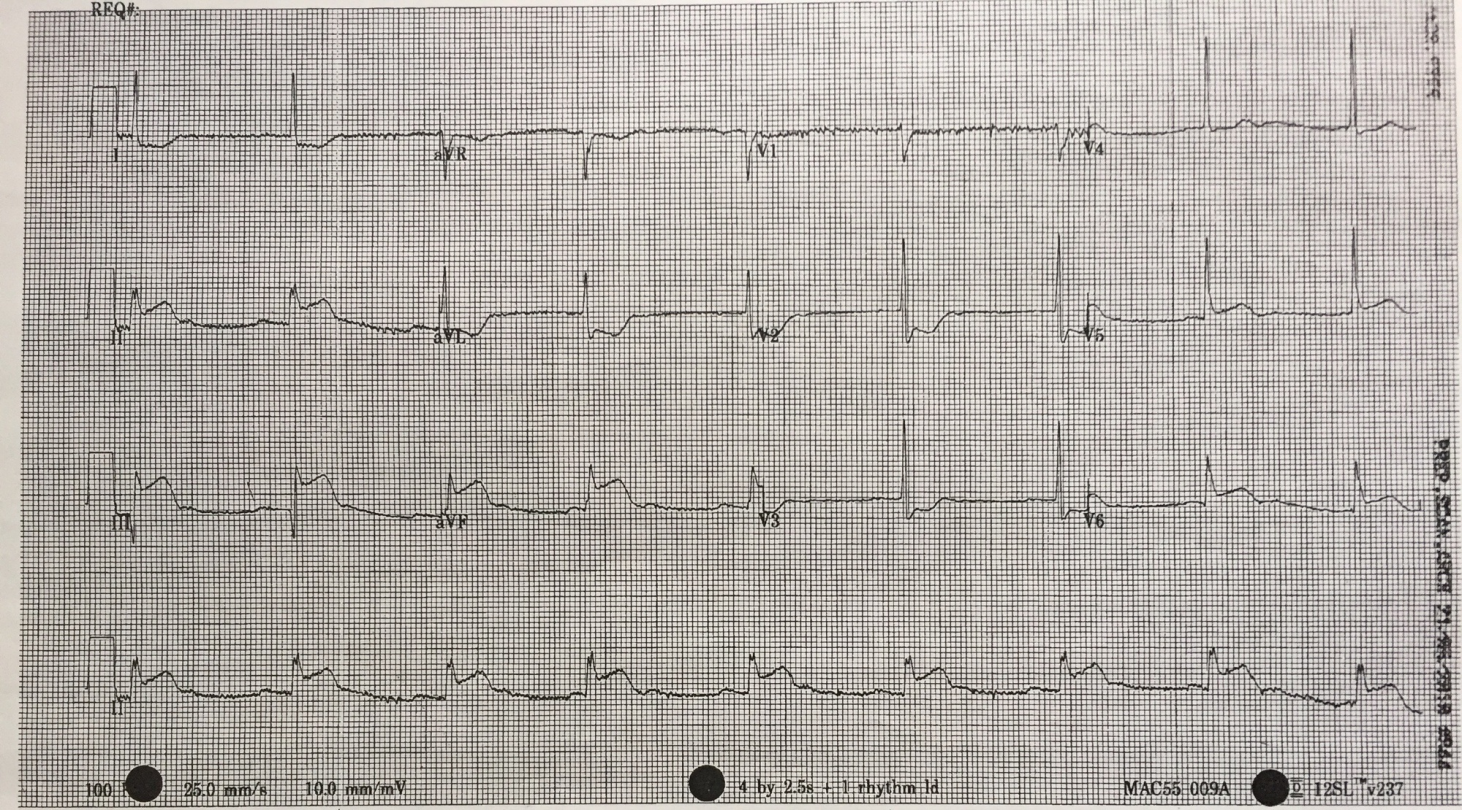
12-lead electrocardiogram (ECG) demonstrating evidence of inferior myocardial infarction (MI). ST-elevation is seen in leads II, III and aVF. Reciprocal changes can be seen in leads I, aVL, V2 and V3. There is also some ST-elevation in leads V5 and V6.

**Figure 3:**
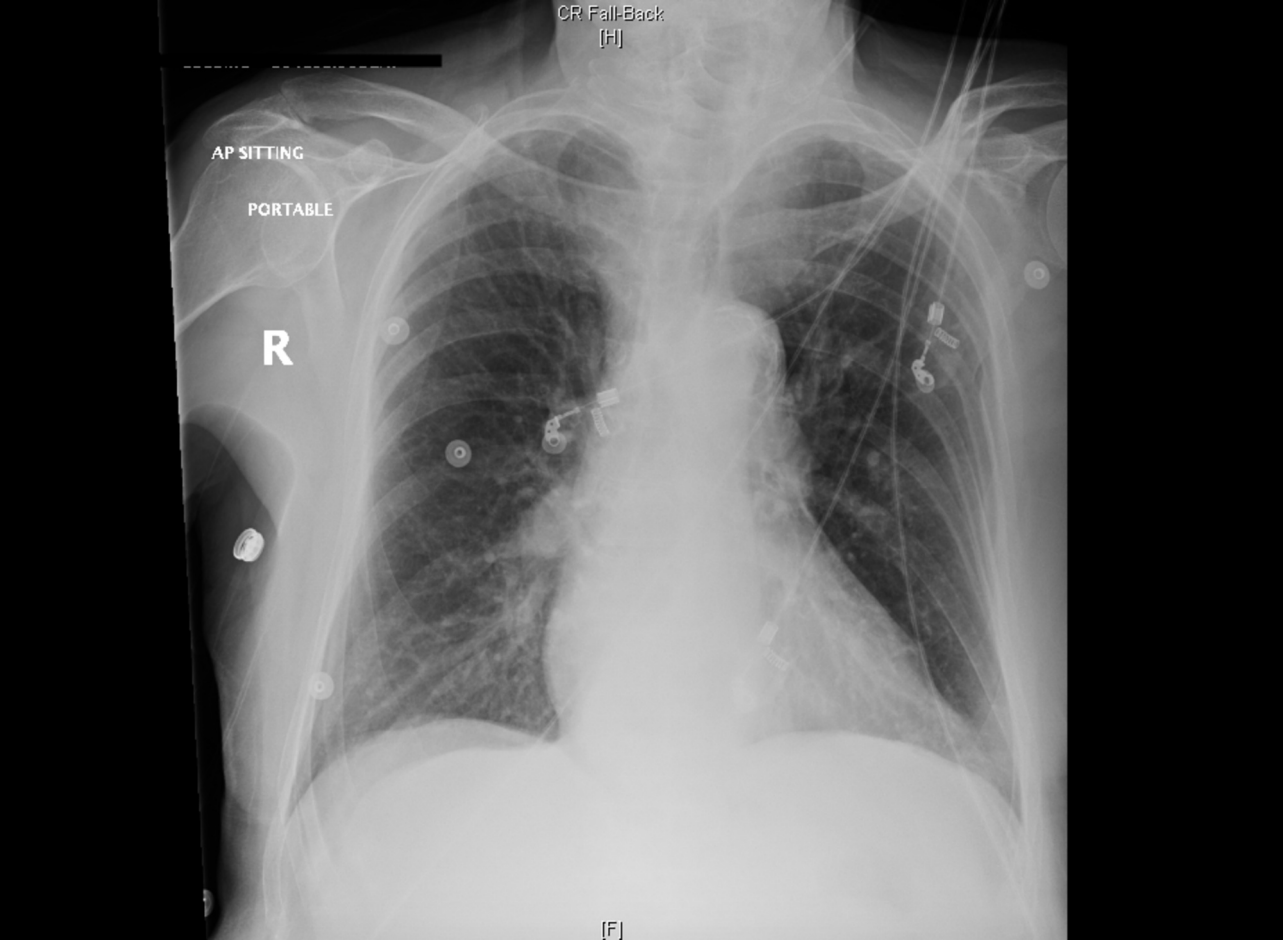
Portable chest X-ray (CXR).

**Figure 4:**
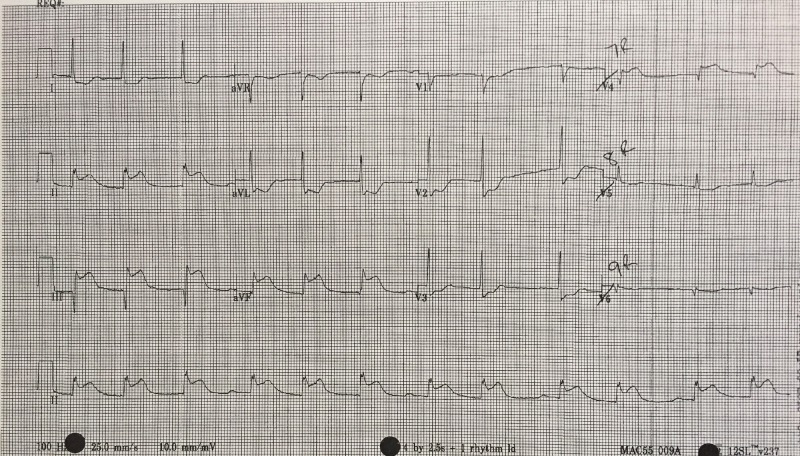
15-lead electrocardiogram (ECG) showing evidence of an inferior myocardial infarction (MI) with right ventricular involvement. ST-elevation is seen in V4R in addition to the inferior leads. Reciprocal changes are noted in leads I, aVL, V2 and V3.

**Figure 5:**
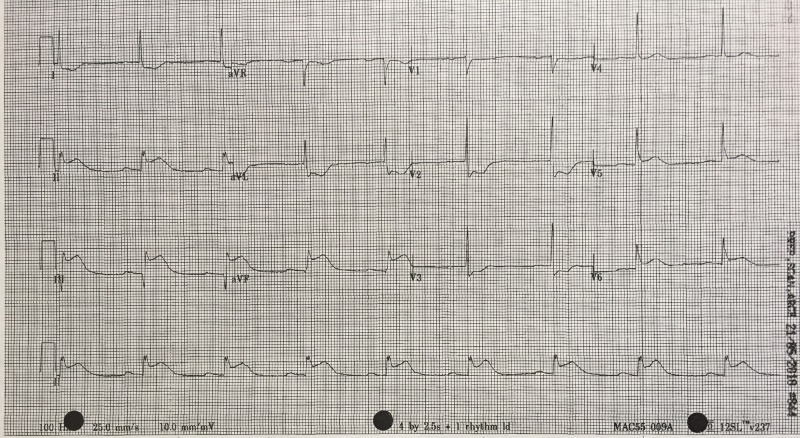
12-lead electrocardiogram (ECG) shortly post fibrinolytic therapy with persistence of ST changes.

**Figure 6:**
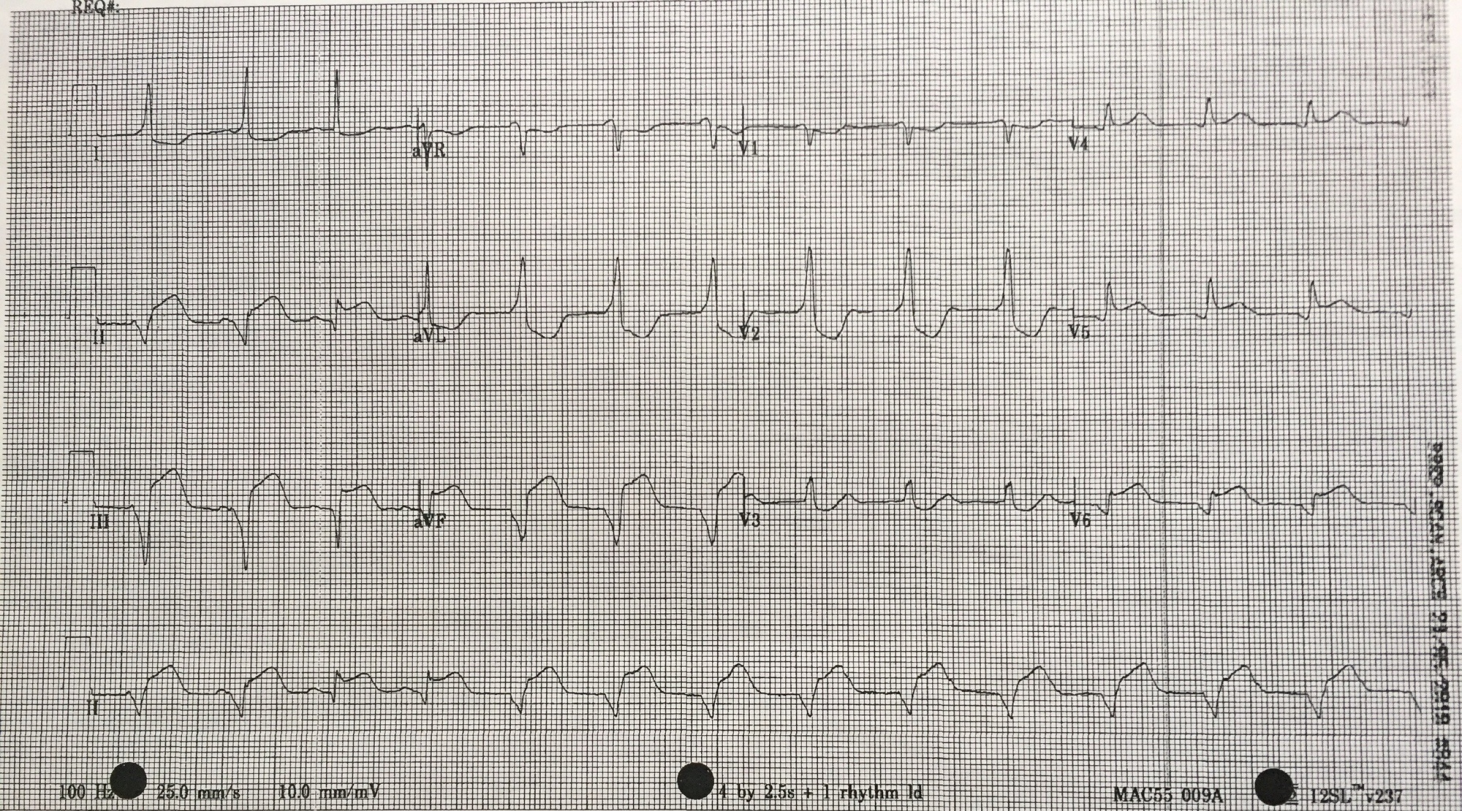
12-lead electrocardiogram (ECG) illustrating accelerated idioventricular rhythm (AIVR) post fibrinolytic therapy.

**Figure 7:**
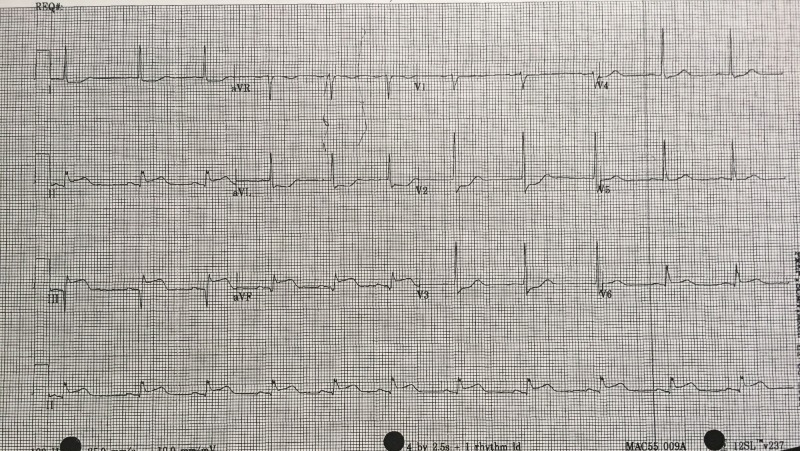
12-lead electrocardiogram (ECG) 90 minutes post thrombolytic therapy showing persistence of ST-elevation in leads II, III, aVF and the associated reciprocal changes.

After a brief introduction to details of the case, trainees are instructed to proceed with the initial evaluation of the patient with supplemental information provided at their request. Table 2 provides applicable laboratory values. Figure 2 is a 12-lead ECG showing an inferior ST-elevation myocardial infarction. Figure 3 is the portable chest X-ray (CXR) for the patient, which has nil acute changes relating to the patient's presentation. Figure 4 is a 15-lead ECG demonstrating evidence of right ventricular involvement, with ST-elevation in lead V4R. The ECG in Figure 5 shows persistence of ST changes after the patient has received thrombolytic therapy. The ECG in Figure 6 demonstrates accelerated idioventricular rhythm (AIVR). This rhythm is often seen some time after administration of thrombolytic therapy and is felt to be associated with reperfusion. Figure 7 is an ECG demonstrating the persistence of ST-elevation later in the case. Table 3 provides an outline of details for an appropriately structured patient handover.

**Table 2:** Relevant laboratory findings to be printed or displayed on screen in simulation lab and provided to trainee when requested.

Laboratory Results	
Complete Blood Count	
White Blood Cell (WBC) Count	9 (x1000/mm^3^)
Hemoglobin (HgB)	128 (g/dL)
Platelets (Plts)	400 (x1000/mm^3^)
Electrolytes, Blood Urea Nitrogen (BUN), Creatinine	
Sodium (Na)	140 (mmol/L)
Potassium (K)	3.8 (mmol/L)
Chloride (Cl)	97 (mmol/L)
Magnesium (Mg)	2 (mmol/L)
Calcium (Ca)	9.5 (mmol/L)
BUN	15 (mmol/L)
Creatinine (Cr)	91 (umol/L)
High Sensitivity Troponin (hsTrop)	
Initial hsTrop	20 (ng/L)
3hr hsTrop	2000 (ng/L)

**Table 3:** Handover information for trainees in the debrief session and to be used for assessment of Objective 5. PMHx: Past medical history; pVT: Pulseless ventricular tachycardia; VF: Ventricular fibrillation; GCS: Glasgow Coma Scale; ACLS: Advanced cardiac life support; CVICU: Cardiovascular intensive care unit.

Handover Item	Completed	Not Completed
Patient Identification		
Name		
Age		
Relevant PMHx		
Diagnosis		
Working Diagnosis (Inferior STEMI with resultant pVT/ VF)		
Corollary Diagnosis as applicable		
Current Status		
Vitals		
GCS		
Interventions		
Medications Administered		
ACLS Protocol Performed (if applicable)		
Lines, tubes, or other adjuncts		
Patient Needs		
CVICU		
Cardiac Catheterization Lab		
Plan and Re-State		
Closed loop communication and clarity of plan		

Debriefing

Following the conclusion of the scenario, the trainees are provided with a formal debriefing. Efforts are made to keep the debriefer-to-learner ratio at 1:1 or as low as possible. This limit encourages learners to speak more freely about challenges they may have faced during the simulation and provides a more comfortable debriefing environment. Depending on the primary faculty running the session, either the advocacy-inquiry model or the 3D model of debriefing can be used [8, 9]. In general we aim to uncover the trainee’s thought process and resulting actions, allowing us to address both errors of process and knowledge gaps to be addressed.

Post scenario didactics

A didactic session is held after the debriefing. This allows instructors to further address knowledge gaps identified through the scenario and debriefing session. Trainees are given the opportunity to reinforce concepts and consolidate knowledge gained through the simulation exercise. The following is an example of various learning needs that may arise following this particular simulation.

1. *Developing a Diagnostic Strategy for Inferior STEMI*

**Figure 8:**
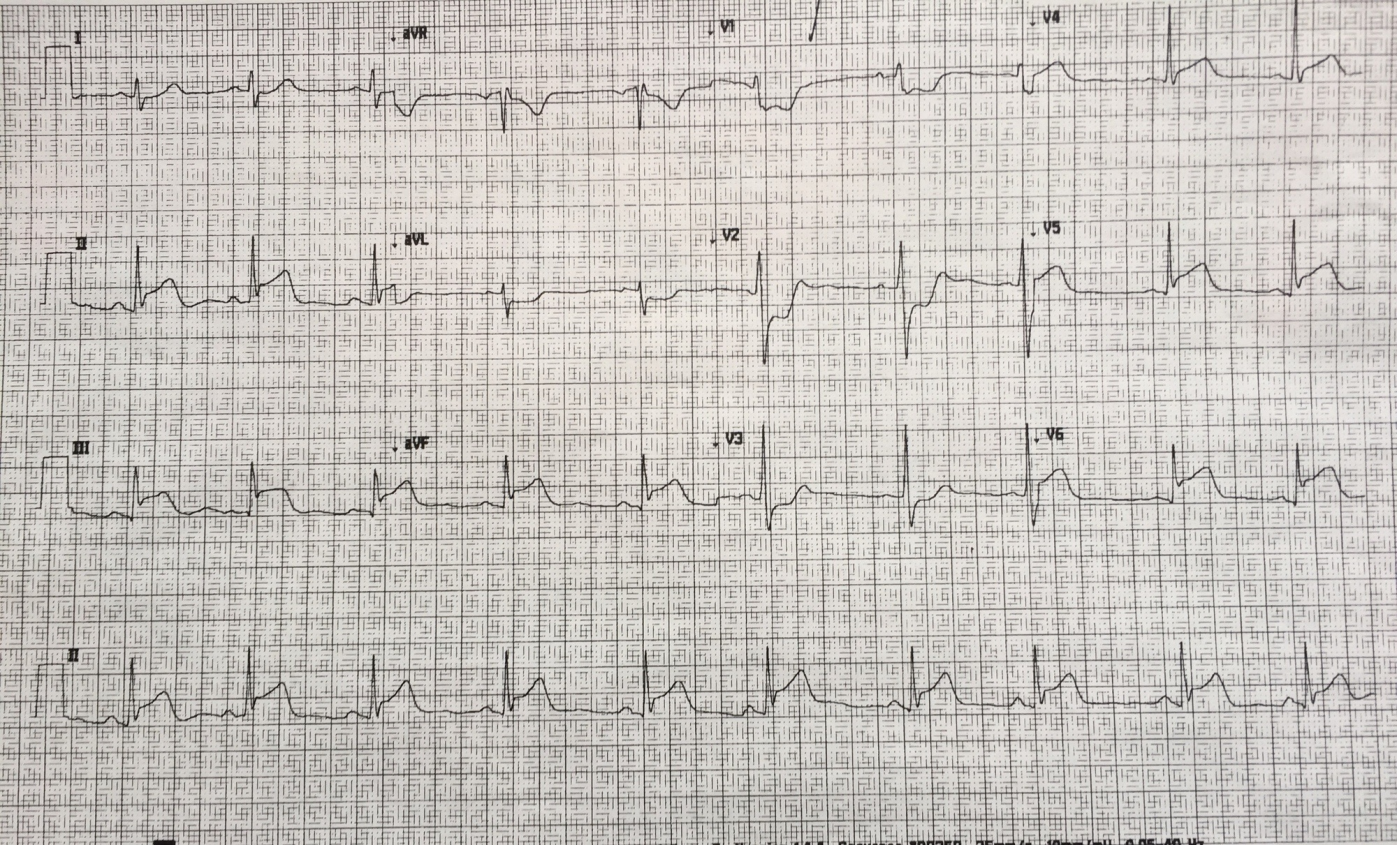
12-lead electrocardiogram (ECG) with evidence of an infero-posterior ST-elevation myocardial infarction (MI) with ST-elevation in leads II, III, aVF in addition to leads V4, V5, V6. There are reciprocal changes in leads I, aVL, V1-V3. Significant ST-depression and tall R-waves are seen in leads V1-V3 that can be an indicator of posterior myocardial damage.

**Figure 9:**
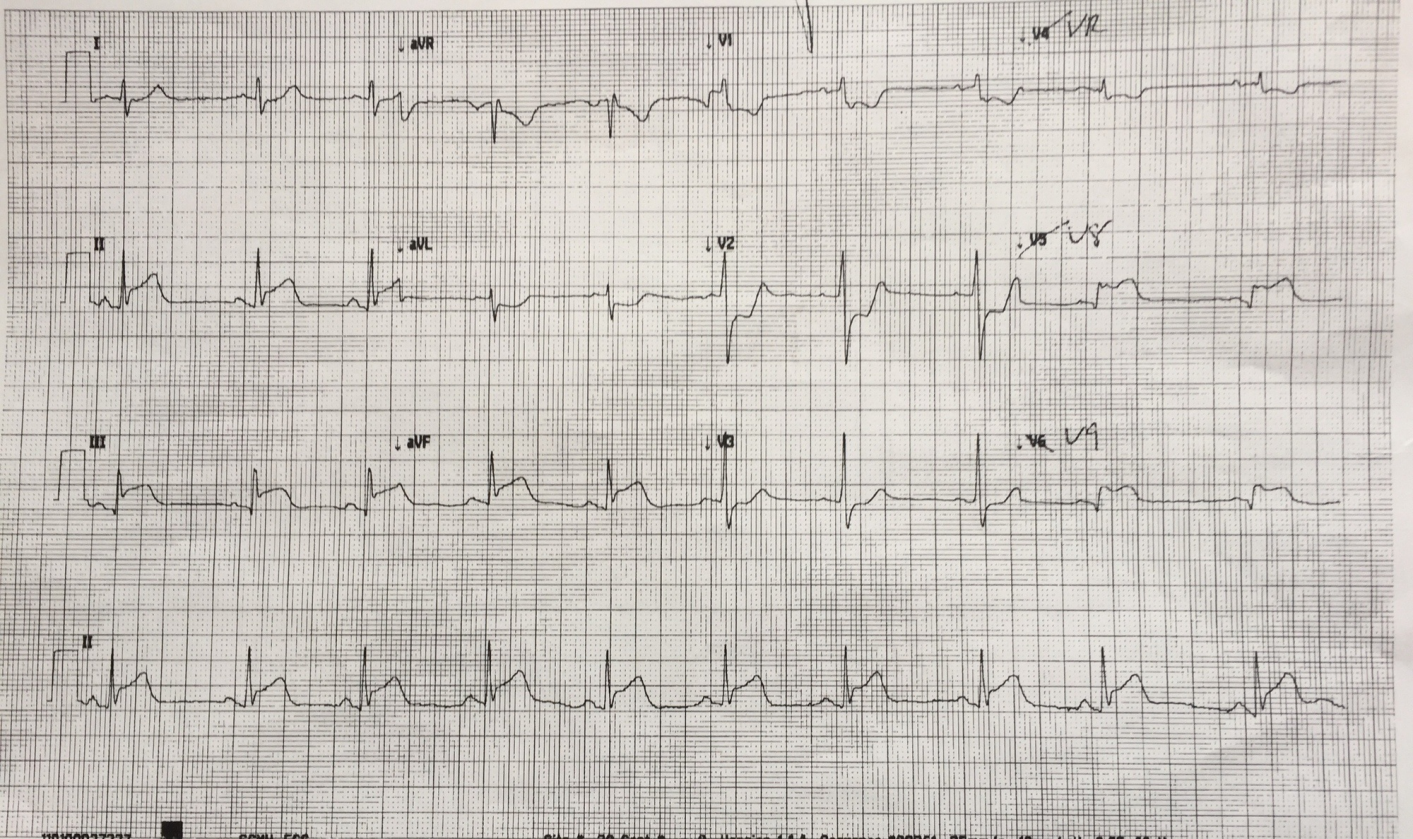
15-lead electrocardiogram (ECG) showing ST-depression in lead V4R and ST-elevation in leads V8 & V9, confirming the presence of a posterior myocardial infarction. This is in addition to the inferior changes in leads II, III, and aVF.

Learners must first determine if patient’s chest pain is likely due to ACS or another cause. A focused history on characteristic presentation of chest pain coupled with a diagnostic 12-lead ECG are used to determine an initial diagnosis of ACS [3]. Further analysis of cardiac biomarker trends is used to confirm the diagnosis, particularly in non-ST-elevation myocardial infarction cases [5]. The ECG appearance of STEMI will vary depending upon the location of coronary artery occlusion and the resultant distribution of myocardium involved. Figure 2 shows the expected ECG changes for the patient in our case of inferior myocardial infarction (MI). For an inferior STEMI with suspected right ventricular involvement, the right precordial leads (V3R, V4R) should be assessed [10]. As visualized in Figure 4, elevation in lead V4R along with inferior changes provides supportive evidence for the diagnosis of concomitant right ventricular (RV) infarction [10, 11]. Identifying this condition is important as it is associated with a higher mortality risk [11, 12]. The presence of prominent R-waves coupled with ST depressions in leads V1-V3 may indicate posterior wall myocardial infarction [2, 3, 7]. Figure 8 shows these changes. A 15-lead ECG is also valuable in identifying posterior infarction as demonstrated in Figure 9 [3]. Without the 15-lead, posterior STEMI may be misdiagnosed due to the absence of “traditional” electrocardiographic infarct signs [13]. The ECGs above are included to be used in the progression of the inferior STEMI case.

2. *Determining a Management Strategy for Inferior STEMI*

Initial therapy prioritizes reperfusion which will result in alleviating chest pain, stabilizing the patient and reducing the symptoms of ischemia [3]. Learners should understand that the choice of reperfusion therapy is primarily dependent on the length of time to access a given reperfusion modality, either thrombolytic or interventional. Primary percutaneous coronary intervention (PCI) is preferred when performed by a skilled interventionalist within 90 minutes of first medical contact at a PCI-capable hospital, or within 120 minutes of first medical contact if transferred to PCI-capable hospital [11, 14]. PCI may also be performed if patient has a high risk of bleeding since fibrinolytic therapy increases the risk of minor and major bleeding conditions. Fibrinolysis is only recommended if PCI cannot occur within 120 minutes, or PCI is not available. Following successful fibrinolysis, subsequent angiography should be performed within 3-24 hours [11, 14].

Integral to the reperfusion modality chosen, patients presenting with an acute myocardial infarction require adjunctive therapies. In the absence of contraindications, these include a loading dose of Aspirin, a platelet aggregation inhibitor (for example, clopidogrel or ticagrelor), and an anticoagulant (such as unfractionated heparin, enoxaparin or fondaparinux). Specific drugs may depend on local availability and preferences as determined in consultation with specialty backup. Complications potentially associated with myocardial infarction, such as bradycardia, hypotension and dysrhythmia, are best treated according to standard Advanced Cardiac Life Support (ACLS) protocols.

**Figure 10:**
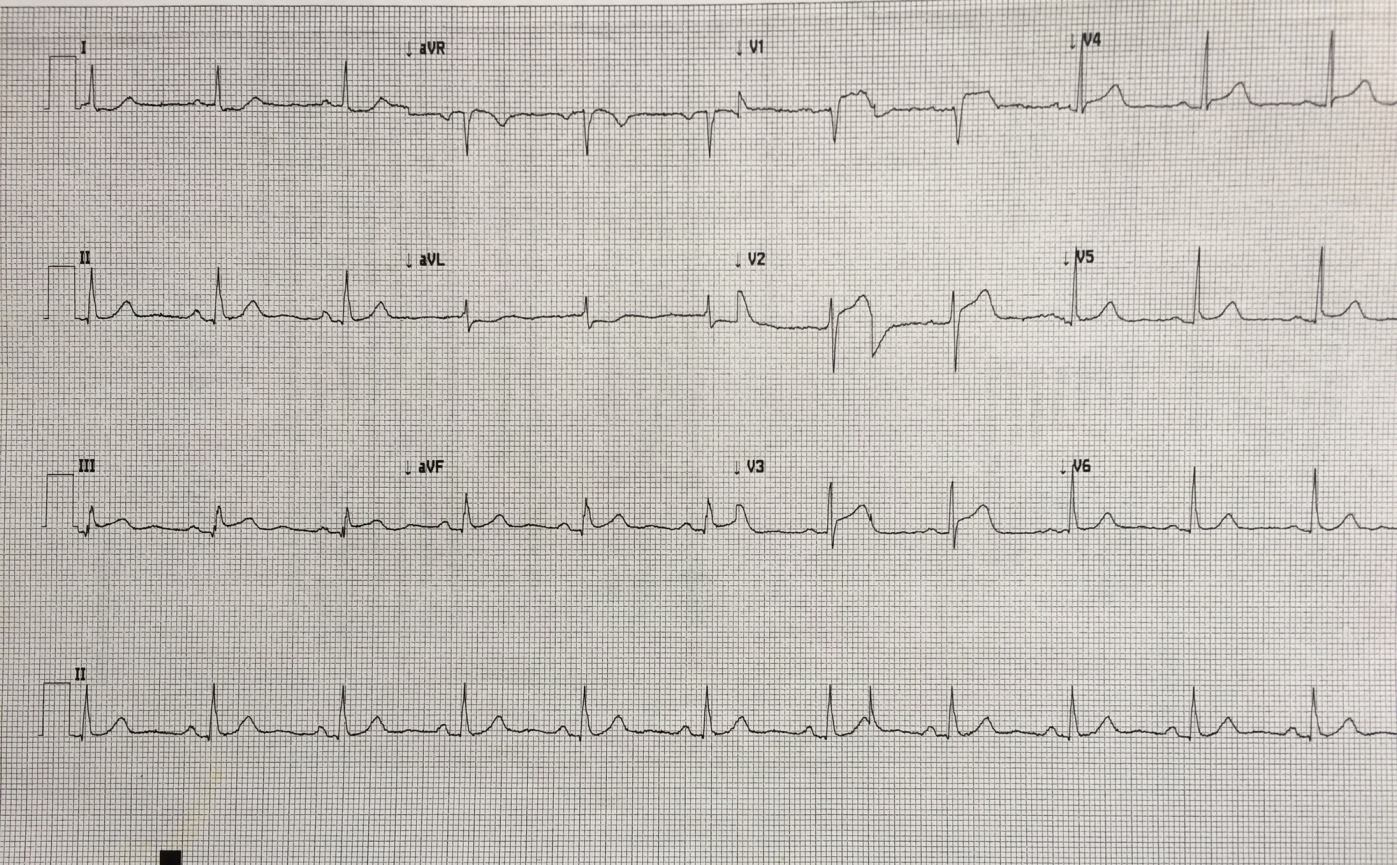
12-lead electrocardiogram (ECG) with evidence of an antero-septal myocardial infarction, with ST-elevation predominantly in leads V1-V3. There is reciprocal ST-depression in leads I and aVL.

**Figure 11:**
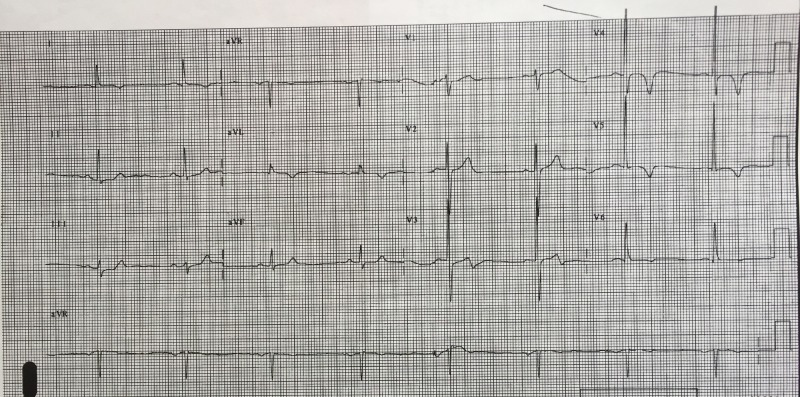
12-lead electrocardiogram (ECG) consistent with Wellens syndrome. The T-wave in lead V3 is biphasic. There are also notable T-wave inversions in leads V4 and V5. The patient was later confirmed to have significant left anterior descending (LAD) occlusion.

**Figure 12:**
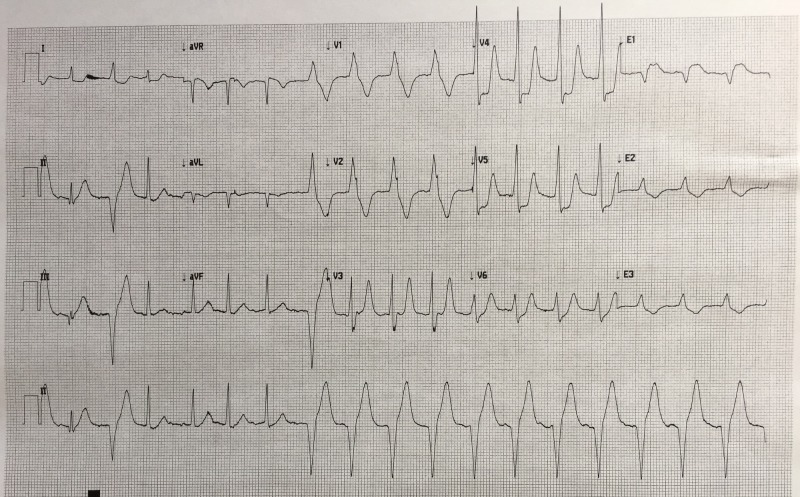
15-lead electrocardiogram (ECG) demonstrating a transition of rhythm from sinus with a PVC into accelerated idioventricular rhythm (AIVR) in a patient administered thrombolytics for the treatment of myocardial infarction.

Three additional ECGs are included in this technical report for the purposes of teaching learners participating in the case. Figure 10 shows ECG findings of an antero-septal myocardial infarct. Figure 11 demonstrates findings consistent with Wellens syndrome which occurs with left anterior descending (LAD) coronary artery disease. Figure 12 provides another example of AIVR. Familiarity with various patterns of ST segment abnormality enables expedient recognition of ECG abnormality in the patient presenting acutely with symptoms concerning for ACS.

## Discussion

The ability to quickly diagnose acute MI is essential to providing life-saving therapy. The goal of this simulation case is to teach the clinical skills necessary to identify and manage the presentation of acute inferior myocardial infarction.

The learning objectives of this case study are:

For the novice learners:

**Objective 1**: Initial management of ACS

**Objective 2**: Making the diagnosis of inferior STEMI and initial management

For the intermediate learners, objectives include the above in addition to:

**Objective 3**: Managing complications

For the advanced learners, objectives include the above in addition to:

**Objective 4**: Managing pulseless rhythms using ACLS

For all learners, the CanMEDS Communicator and Collaborator roles can be incorporated with the following objective:

**Objective 5**: Structuring a patient handover

The stepwise algorithm allows the simulation to be flexible and adaptable to different levels of learner. The simulation scenario dry-run enables an instructor to assess, identify, and address difficulties with the case prior to this session. Post-session formal debriefing, coupled with didactic teaching, allows instructors to discuss performance during the simulation and address learning needs that arise during the process.

This simulation is intended to train different levels of learners, including novice, intermediate and advanced. There are three stages of progressing difficulty, with suggested end-points at each level. Instructors will use an Entrustable Professional Activities (EPA) assessment tool to evaluate trainees during the simulation. At the suggested end-points, trainees may advance on to the next level of the simulation based on their performance. If trainees do not satisfy expectations at their expected level of performance, the formal debriefing and post-didactic session will provide an opportunity to identify errors of process and knowledge gaps. The design structure of the case allows it to be applied with varying degrees of competencies, from medical students to emergency medicine residents. Each simulation may be tailored to match a trainee's ability, to provide an optimal learning experience. Furthermore, this design facilitates the continual development of competencies. The debriefing and post-didactic sessions should improve the trainee’s approach to inferior STEMIs, allowing for advancement in future trials. A number of additional ECGs to be used as resources for discussing ECG findings associated with different pathology including posterior MI (Figure 8), posterior lead ST-elevation (Figure 9), antero-septal MI (Figure 10), Wellens syndrome (Figure 11), and an additional example of AIVR (Figure 12) are included in the paper above.

With the ongoing shift to competency-based training in post-graduate medical education, using a layered approach is not only important, but timely as well. Resident physicians in Canada are now being evaluated based on their competence at various levels of training, similar to the approach taken in this simulation exercise. Using layers of difficulty in simulation can map well to increasing level of competency required by post-graduate learners as they progress towards independent practice. This scenario, and others with a similar style could be used longitudinally with increasing difficulty as learners become more proficient, similar to adding (EPAs) to a learner's acumen as they advance in their training.

This simulation was designed to familiarize trainees with the proper diagnosis and management of ST-elevation myocardial infarction, in the emergency medicine setting. As such, the therapies taught are limited to acute interventions. Further management of the patient’s condition requires consultation with other medical specialists, chiefly Cardiology. This provides the opportunity for learners to develop skills in competencies including collaboration and communication. Specifically, there is an opportunity for learner(s) to discuss the case with an interventional cardiologist via telephone, or face-to-face with an on-site medicine specialist. Trainees were expected to identify when consultation is necessary during the simulation, with a particular focus on reperfusion modalities relating to their practice location [3, 11, 14]. These moments allowed trainees to foster their collaborative skills by interacting with other healthcare professionals. The overall goal is the provision of high-quality, patient-centred care.

## Conclusions

Prompt recognition of inferior STEMI is essential as it directly influences the type and efficacy of reperfusion therapy. This is a teaching session on inferior STEMI that incorporates simulation learning with debriefing and didactic components. In an effort to better match this scenario to potential competency-based post-graduate programs, we used a layered approach which allows learners at varying stages to have different levels of difficulty. In addition, we incorporated the CanMEDS roles of medical expert (case completion) and collaborator/communicator (handover and consultation with a cardiologist).

## Appendices

APPENDIX 

**Table 4:** Complete list of Abbreviations

Abbreviations	Description
GERD	Gastroesophageal Reflux Disease
MI	Myocardial Infarction
JVD	Jugular Vein Distension
ECG	Electrocardiogram
BP	Blood Pressure
RR	Respiratory Rate
SpO_2_	Oxygen Saturation
IV	Intravenous
ASA	Acetylsalicylic Acid
STEMI	ST Elevated Myocardial Infarction
WBC	White Blood Cell
HgB	Hemoglobin
Plts	Platelets
Na	Sodium
K	Potassium
Cl	Chloride
Mg	Magnesium
Ca	Calcium
BUN	Blood Urea Nitrogen
Cr	Creatinine
hsTrop	High Sensitivity Troponin
